# Optimal two-time point longitudinal models for estimating individual-level change: Asymptotic insights and practical implications^☆^

**DOI:** 10.1016/j.dcn.2024.101450

**Published:** 2024-09-24

**Authors:** Andreas M. Brandmaier, Ulman Lindenberger, Ethan M. McCormick

**Affiliations:** a Department of Psychology, MSB Medical School Berlin, Germany; b Center for Lifespan Psychology, Max Planck Institute for Human Development, Germany; c Max Planck UCL Centre for Computational Psychiatry and Ageing Research, Germany; d Methodology and Statistics Department, Leiden University, The Netherlands

**Keywords:** Latent change score model, Latent curve model, Reliability, Precision, Individual differences, Latent difference score model, Longitudinal model

## Abstract

Based on findings from a simulation study, Parsons and McCormick (2024) argued that growth models with exactly two time points are poorly-suited to model individual differences in linear slopes in developmental studies. Their argument is based on an empirical investigation of the increase in precision to measure individual differences in linear slopes if studies are progressively extended by adding an extra measurement occasion after one unit of time (e.g., year) has passed. They concluded that two-time point models are inadequate to reliably model change at the individual level and that these models should focus on group-level effects. Here, we show that these limitations can be addressed by deconfounding the influence of study duration and the influence of adding an extra measurement occasion on precision to estimate individual differences in linear slopes. We use asymptotic results to gauge and compare precision of linear change models representing different study designs, and show that it is primarily the longer time span that increases precision, not the extra waves. Further, we show how the asymptotic results can be used to also consider irregularly spaced intervals as well as planned and unplanned missing data. In conclusion, we like to stress that true linear change can indeed be captured well with only two time points if careful study design planning is applied before running a study.

## 1 Introduction

Latent Curve Models (LCM) and Latent Change Score Models (LCSM) have become standard techniques to model individual differences in change over time (Grimm et al., 2012, Kievit et al., 2018, McCormick et al., 2023). In these models, the latent factors represent both group-level and individual differences in some assumed shape of change (e.g., a difference, a linear function, a quadratic curve) over multiple occasions of measurement. Modeling individual differences in change is of central interest in developmental studies spanning the lifetime because humans differ in their rates of change across various domains of functioning (e.g., cognitive, motor, or affective) and levels of analysis (e.g., behavioral or neural) (Lindenberger, 2014). To appropriately model these trajectories and advance scientific theory, statistical models of change must have adequate precision to measure both mean change and individual differences in change. Typically, statistical power – that is, the probability of finding a hypothesized effect if it really exists – is considered as the primary measure of precision. In addition, more general metrics to gauge the sensitivity of latent models for individual differences in linear change are available, such as effective error variance and effective curve reliability (Brandmaier et al., 2018, Rast and Hofer, 2014, Willett, 1989).

Linear latent change score models are often used as a parsimonious approach to estimate an average gradient of change, typically referred to as linear *slope*, as well as individual differences in the linear slope across persons. Even if true change is non-linear, they often serve as useful tools to linearly approximate change in a given time window (but see Ghisletta et al. 2020). At least to a limited degree, they also allow for modeling non-linear change if the dependent variable or the timing variable is transformed using a non-linear transformation, such as the logarithm or the square-root. These models are becoming increasingly prevalent within the field of developmental cognitive neuroscience, and primers on latent change scores and latent curve models have recently made these methods much more accessible (Kievit et al., 2018, McCormick et al., 2023), including code for fitting them in practice. For specific reference to the two time point model, see the explication by Parsons and McCormick (2024).

In a recent article, some of us (Parsons and McCormick, 2024) offered a critique of current practices for modeling longitudinal data with relatively few measurement occasions, especially in relation to the recent increase in two-time point models using data from the Adolescent Brain Cognitive Development [ABCD; Casey et al. (2018)] study. Parsons and McCormick (2024) investigated the precision with which individual difference scores (using LCSMs) and slopes (using linear and quadratic LCMs) can be estimated. As a metric for the precision of the model, Parsons and McCormick (2024) proposed the correlation of the estimated individual slopes and the true slopes. They found that the correlation was quite poor for the two-time point models considered, and the correlation increased with every measurement occasion added to the model. From this observation, they concluded that two-time-point models are poorly suited to model *individual differences* in slopes in developmental psychology, as the shared variance between true change scores and estimated change scores was low (16.8% in their simulation conditions), although they highlight that other features, such as mean change, can be captured more reliably.

Parsons and McCormick (2024) paint a relatively gloomy picture for models with two time points as they are typically used — that is, the first two measurement occasions of a longitudinal study that are relatively closely spaced in time (most often on an annual or biannual basis). However, it is theoretically and practically possible to design studies with high precision to estimate linear change with only two time points if we are willing to depart from these typical use-cases. Here we lay out strategies for doing so. Our main argument is two-time point models are often inadequate because the time elapsing between measurements is simply too short in relation to the development of individual differences in linear slopes. To answer the question whether two-time point models are generally inadequate in capturing individual differences in change, we need to systematically vary (i.e., unconfound) the number of measurement occasions from the time elapsing between measurements. In the remainder of this manuscript, we will show how asymptotic estimates of precision can be leveraged to gauge and compare the precision of different study designs analyzed with linear latent change score models. From these results, we can see that the effect of total study time on precision is quadratic and can be more influential than the number of measurement occasions, especially in longitudinal designs with low measurement frequency (e.g., less than five measurement occasions). In such cases, the increase in statistical power by adding another measurement largely reflects the increase in study duration rather than the addition of another observation. In the remainder, we demonstrate how a principled understanding of study duration and number of measurement occasions can guide the design of studies that make optimal use of scarce resources (e.g., measurement occasions) in achieving precision and reliability of the estimated effects (also see Brandmaier et al. 2015).

For simplicity of our argument, we deviate slightly from the main model specification suggested by Parsons and McCormick (2024). They chose measurement error variance at each occasion such that the variance explained by latent intercept and slope is 50% of the total observed variance at every time point. However, this corresponds to a measurement instrument that becomes systematically less reliable over time, which we argue is not the most common case in longitudinal studies span multiple years (yet, systematic influences on reliability may arise be due to participants growing acclimated to the scanner or bored with the experiment). For example, if we assume that we investigate training-related gains (say, in episodic memory performance) in a training study, then intercept variance in the LCM corresponds to the individual differences in memory performance at study onset. Parsons and McCormick (2024) set the residual error variance at $\sigma_{e}^{2}=1$, thus reliability of the measurement instrument at the first wave is 0.5. After five years, the variance explained by intercept and slope and the residual error each are $1+2\cdot 5\cdot 0.15+5^{2}\cdot .25=8.75$ (assuming an intercept-slope-correlation of 0.15 and a slope variance of 0.25). That is, after five years, the measurement instrument is assumed to only have a reliability of $\frac{1}{1+8}\approx 0.10$. In the remainder, we deviate from this and assume a measurement instrument with constant reliability over time, consistent with the sensitivity analysis presented in the Supplemental Code (https://osf.io/9rjcv/) provided by Parsons and McCormick (2024).

To evaluate the precision with which a latent construct can be measured given a particular longitudinal study design (e.g., number and timing of observations), we can rely on the notion of *effective error* (Brandmaier et al., 2018, von Oertzen and Brandmaier, 2013). Effective error is an asymptotic estimate of the measurement error for a given measurement instrument used to assess a latent construct. The (inverse of the) variance of the effective error gives a valid measure of precision that, together with ideas from classic test theory, can be used to derive a reliability measure for latent variables (Brandmaier et al., 2018), making it a useful tool to compare precision across a wide array of study design conditions. von Oertzen and Brandmaier, 2013, Brandmaier et al., 2015 developed the asymptotic equations for the effective error of the linear slope in linear latent growth models. For a linear latent growth model with M observations that occur at time points $t_{1},t_{2},\ldots ,t_{M}$, an intercept variance $\sigma_{I}^{2}$, and a residual error variance $\sigma_{E}^{2}$, and no intercept-slope-correlation, the effective error variance is:

(1) $$\sigma_{eff}^{2}=\frac{\sigma_{E}^{2}}{\sum \overset{2}{\underset{i}{t}}-\overset{2}{\underset{I}{\sigma }}/\left(\sigma_{I}^{2}\cdot M+\sigma_{E}^{2}\right)\cdot {\left(\sum t_{i}\right)}^{2}}$$

As we can see, the precision with which we can estimate linear slopes scales with the residual error (that is, the inverse of the precision) of the measurement instrument used at every wave ($\sigma_{E}^{2}$), and quadratically depends on total study time $t_{M}$. Similar but more complex solutions for models with non-zero intercept-slope-correlation exist (Brandmaier et al., 2018).

## 2 Precision of individual differences in linear slopes

Brandmaier et al., 2015, Brandmaier et al., 2018 proposed effective curve reliability (ECR) to gauge the sensitivity of a growth model to measure individual differences in linear slopes (represented as the variance of the latent linear slope variable of a LCM). ECR is the ratio of true-score variance, here the slope variance $\sigma_{S}^{2}$ to the sum of true-score variance and error variance, here $\sigma_{eff}^{2}$. ECR is an estimate of the slope reliability, which ranges between 0 and 1, with higher values indicating higher reliability. This interpretation follows from classical test theory and can be understood as the reliability of the slope as if the slope were measurable with a single observation. For a given sample size and significance level, ECR directly translates to statistical power of hypotheses about the slope. Importantly, we can derive the asymptotic correlation of the estimated slopes and true slopes, which was proposed as a measure of precision by Parsons and McCormick (2024), directly from ECR. Consider a model with two observed variables, one is the true score (with variance $\sigma_{t}^{2}$) and one is a true score that is a noisy observation of the true score (with error variance $\sigma_{e}^{2}$). From this model, we can derive the covariance matrix of those two variables (representing the noisy observation and the true score):

(2) $$\left[\begin{array}{cc} \sigma_{t}^{2}+\sigma_{eff}^{2} & \sigma_{t}^{2}\\ \sigma_{t}^{2} & \sigma_{t}^{2} \end{array}\right]$$

Given that the true scores represent true linear slopes, the upper right element (or, by symmetry, the lower left element), $\sigma_{t}^{2}$ corresponds to the covariance of true slopes and estimated slopes. The upper left element is the variance of the slopes estimated from a LCM or LCSM, and the lower right element is the variance of the true slopes. From this, we can derive the correlation of true slopes and estimated slopes using the well-known transformation of a covariance into a correlation as:

(3) $${\overset{ˆ}{\rho }}_{x,\overset{ˆ}{x}}=\frac{\sigma_{t}^{2}}{\sqrt{\sigma_{t}^{2}}\cdot \sqrt{\sigma_{t}^{2}+\sigma_{eff}^{2}}}$$

which we can simplify further to

(4) $${\overset{ˆ}{\rho }}_{x,\overset{ˆ}{x}}=\sqrt{\frac{\sigma_{t}^{2}}{\sigma_{t}^{2}+\sigma_{eff}^{2}}}=\sqrt{ECR}$$

This connection allows us to asymptotically compute the precision of individual slope estimates as proposed by Parsons and McCormick (2024) without the need to resort to simulation-based approaches. It provides us with means to evaluate and compare different linear latent growth curve models in terms of the correlation of true individual linear slopes and estimated individual linear slopes.

**Fig. 1:**
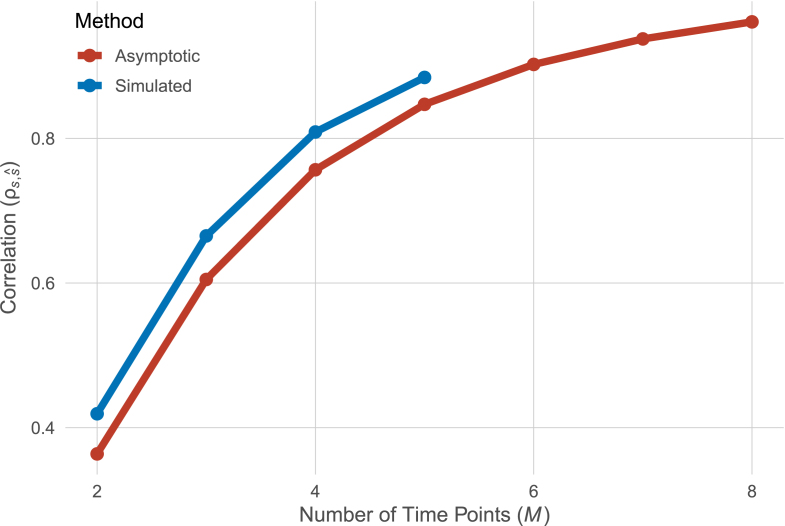
Asymptotic (red) and simulated (blue) correlations of true slopes and estimated slopes. (For interpretation of the references to color in this figure legend, the reader is referred to the web version of this article.)

To illustrate, we first evaluate the asymptotic correlation for the model proposed by Parsons and McCormick (2024). They defined a latent covariance matrix of intercept and slope (co)variances:

(5) $$\Psi =\left[\begin{array}{cc} 1 & 0.15\\ 0.15 & 0.25 \end{array}\right]$$

with equally spaced measurement occasions at every unit of time (e.g., years). As mentioned before, Parsons and McCormick (2024) chose a model in which measurement error increases as a function of time. Here, we assume that reliability of the measurement instrument (e.g., a magnet resonance [MR] scanner or a questionnaire) is stable over time. We choose a residual error variance of 1, which corresponds to the residual error variance chosen by Parsons and McCormick (2024) at study inception.

Given these assumptions, effective error variance can now be easily computed for different study designs. To illustrate, effective error variance for a three-time point model can be computed by substituting the assumptions about true model parameters into Eq. (1) (for simplicity of the argument, assuming no intercept-slope covariance for now):

(6) $$\sigma_{eff}^{2}=\frac{1}{\left(0^{2}+1^{2}+2^{2}\right)-1/\left(1\cdot 3+1\right)\cdot {\left(0+1+2\right)}^{2}}=0.3125$$

thus, ECR is

(7) $$ECR=\frac{0.25}{0.25+0.3125}=0.44$$

Using our earlier result (Eq. (4)), we obtain an asymptotic correlation of true scores and estimated scores of $\sqrt{0.44}=0.66$. Fig. 1 shows a comparison of the asymptotic and simulated values based on this model where the number of time points is varied between 2 and 8 (the original simulation only varied between 2 to 5 time points).As can be seen, the asymptotic results closely match the simulated results.

### 2.1 Maximizing the utility of only two time points

The poor performance of the two-time point model assessed in Parsons and McCormick (2024) reflects the combination of two separable factors: a low number of measurement occasions (i.e., two), and a short duration of the study. Their resulting critique of two-occasion models was targeted at secondary data analyses of large on-going studies of developmental change, where this confounding is brought about by the sequential release of available data (e.g., as subsequent measurement occasions are being completed). . In these designs, and in the critique by Parsons and McCormick (2024), adding a measurement occasion always increases the total study time span by roughly one unit of time, thereby producing a complete confound between study duration and number of occasions. However, this confound is by no means inevitable. Instead, on the basis of Eq. (1) and for a given ECR, we can plan longitudinal studies that optimize the relation between these two design parameters to detect variance in change (Brandmaier et al., 2015).

**Fig. 2:**
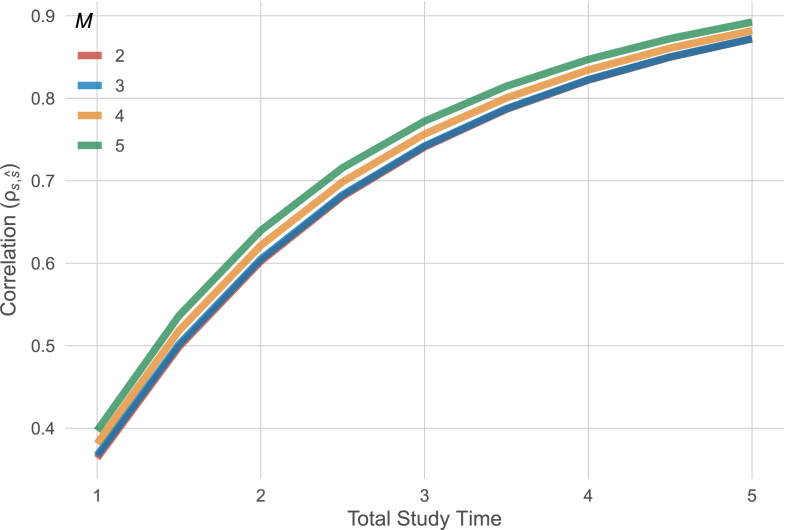
Precision of slope estimates as a function of time and number of measurement time points. The two-time point and three-time point models have almost identical precision.

From Eq. (1), we can infer that the effect of time on ECR is asymptotically quadratic, that is, increasing total study time has typically a larger effect than adding a measurement occasion when keeping total study time constant. In earlier work, we have illustrated this effect in an example inspired from a memory task in the Berlin Ageing Study (BASE). This study had 6 waves over 13 years. von Oertzen and Brandmaier (2013) showed that a study with only 5 waves would only have to last 12 days longer to have identical statistical power. In other words, by removing an entire wave from the study virtually no precision or statistical power to detect individual differences in linear slopes was lost. Using the asymptotic results from above, we can investigate the effect of study duration and number of measurement time points for the model proposed by Parsons and McCormick (2024). Fig. 2 shows the asymptotic correlation of true slopes and estimated slopes as a function of both time and number of equally-spaced time points. We can see that with increasing study duration, the correlation increases. Importantly, for a given study duration, equally distributing more waves in the same amount of time, has hardly an effect on the correlation. This is because the effect of total study time is quadratic in the precision and dominates the terms that arise from adding measurement occasions (see Eq. (1)). Vice versa, a two-time point model with one wave at study onset and one wave after four years has almost identical precision to a study design in which there are yearly waves over four years. This suggests that two-time point models can be useful for understanding individual differences in linear slopes when we deviate from the default longitudinal design of annual observations. Here we refer to annual observations given the wide prevalence of this approach in developmental research. However, in some applications, annual observations might represent quite long “effective” study duration. In all cases, study duration needs to be contextualized by the time course of the phenomenon of interest.

### 2.2 Optimal design

Given an asymptotic result for the precision of a LCM or LCSM, we should ask ourselves: What is the optimal design to capture individual differences in linear slopes (Brandmaier et al., 2015)? As argued earlier, there are different possible metrics for assessing the precision of capturing change depending on whether we would like to consider specific choices about sample size, significance level or not (see example #2 of Brandmaier et al. 2018 for an illustration). For now, we focus on ECR as a measure of reliability (assuming that sample size is fixed at some determined value and significance level also remains at a fixed value, say α=0.05).

Under simplified conditions, effective error asymptotically depends on the variance of the time points, a result that was already found by Willett (1989). For example, in a five-wave linear LCM with equally spaced measurement intervals at 1 unit of time, the time points are 0,1,2,3 and 4. The mean of the time points is 2 and the variance is $\left({\left(0-2\right)}^{2}+{\left(1-2\right)}^{2}+{\left(2-2\right)}^{2}+{\left(3-2\right)}^{2}+{\left(4-2\right)}^{2}\right)/5=2.5$. The larger this variance, the lower the effective error, the larger the reliability and hence the larger the correlation of true scores and estimated scores. When is variance maximal and thus reliability optimal? Variance is (asymptotically) at its maximum if we assign the measurement occasions equally to the study onset and study end. For example, if we could afford six measurement occasions over three years, variance across time points is maximal if our time points are (0,0,0,3,3,3), that is, we measure three times in a row at the beginning of a study (say, repeat the same MR sequence three times without removing the person from the scanner) and the same three times after three years, similar to measurement burst designs (Stawski et al., 2015). As a consequence, the six time-point model really converges to a two-time point latent change score model with multiple indicators. We can conclude that linear change is measured best with two time points that are measured well. The geometric intuition behind this is that a line is defined by two points and it is sufficient to measure these two points well (e.g., by repeated measures very close in time). This means that the two-time point latent change score model (with multiple indicators) has the potential to be the optimal model for assessing individual differences in linear slopes (if the model assumptions are correct!), whereas common developmental designs, as assessed by Parsons and McCormick (2024), represent the worst case for reliability in a two-time point model. In practice, optimality is a function of resource needs and costs, which can be formally included in considering optimal designs (Brandmaier et al., 2015).

### 2.3 Considerations for optimizing two-time point models

While designs like the (0,0,0,3,3,3) approach outlined above offer the chance to maximize the reliability of two-time point models, there are some potential considerations we need to be aware of. Here we outline two: (1) the role of planned and unplanned missing data, and (2)nonlinear functional forms.

#### 2.3.1 Planned and unplanned missing designs

**Fig. 3:**
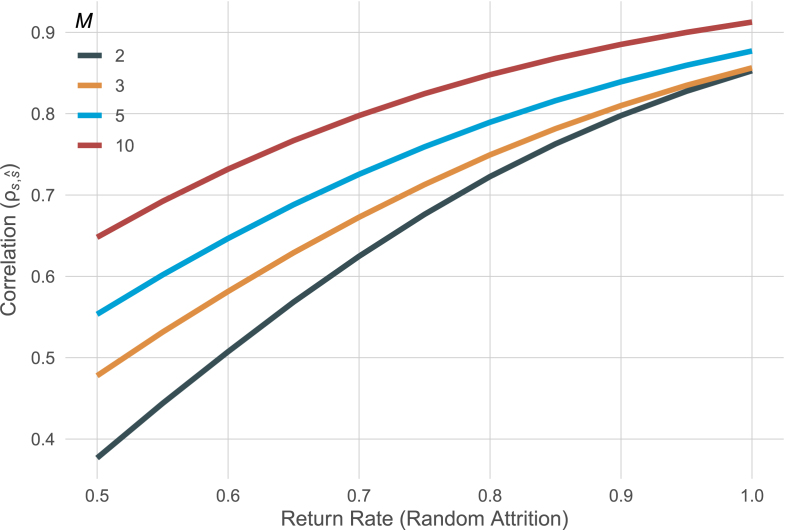
Asymptotic effect of missing data on the precision of individual differences in linear slopes for study designs with fixed total study time (of four years) and varying number of measurement occasions (M).

A particular challenge in the practical implementation of longitudinal studies is the fact that not all participants can be measured at later measurement occasions. The reasons are manifold. For example, later measurements may be missing because people move, lose interest in the study or can no longer participate in the study for other reasons. Typically, the likelihood of a person not returning increases with the length of the study, and any such person with missing measurement points naturally brings less information about their change. Therefore, when considering optimal designs for change measurement, we are dealing with two opposing forces. The longer we wait, the more accurately we can measure individual differences in linear slopes, but the more likely we are to lose subjects and thus power.

von Oertzen and Brandmaier (2013) have worked out the asymptotic effective error for observations missing completely at random. To consider random attrition, they showed that one first computes effective error for each pattern of missing data (for example, the effective error of a five wave design, in which the fourth occasion is missing, another five wave design, in which the third occasion is missing, and so on) and then computes the weighted harmonic mean of these effective errors (see their Eq. (5)). Using this result, we can compute the asymptotic correlation of true scores and estimated scores based on an assumed missing data mechanism. von Oertzen and Brandmaier (2013) reported a return rate in the BASE study of about 83% per year. That is, if N participants are observed at study onset, after k years, we expect that $0.83^{k}\cdot N$ participants are observed. In this example, if there were 200 participants in the first wave, we expect that only $0.83^{2}\cdot 200\approx 137$ participants return after two years. Fig. 3 illustrates the asymptotic effects of attrition for different return rates for the model of our previous example (again, assuming negligible intercept-slope-covariance). We can see that under complete data, the two and three time point models have identical precision, the three time point model loses less precision under missing data because it can still use data from people who returned in the middle of the study. Further, the more waves in a study, the smaller the loss in precision due to high attrition rates.

Brandmaier et al. (2020) used this result to show how, under assumptions of random attrition, we can optimize precision of linear latent growth models to detect individual differences in linear slopes if we want to employ planned missing data designs, that is, the deliberate omission of entire waves for randomly selected participants. This approach has the potential to save resources while guaranteeing adequate statistical power.

#### 2.3.2 Nonlinear trajectories

**Fig. 4:**
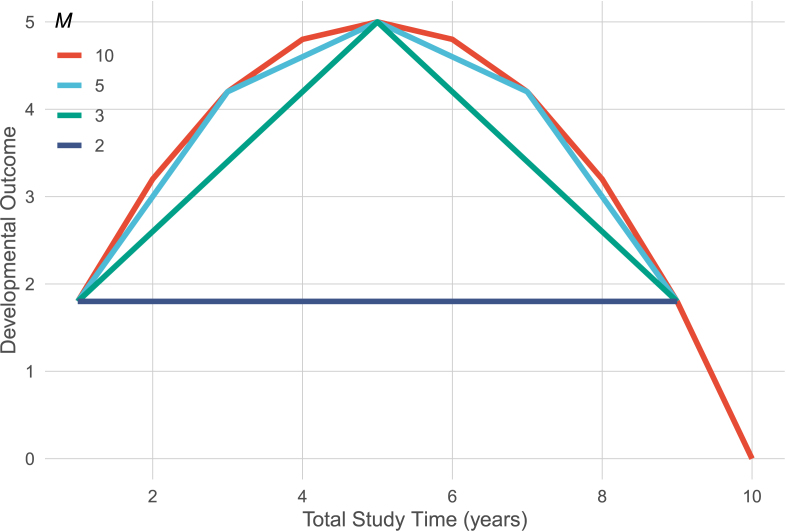
Extending the interval between measurement occasions (1 year interval in red to 9 year interval in purple) can reduce the ability of lower time point models to approximate nonlinear change. (For interpretation of the references to color in this figure legend, the reader is referred to the web version of this article.)

Another opposing force that complicates decisions of whether to wait for longer intervals between measurement occasions is that the asymptotic derivations of effective error and ECR we outline assume that change is linear across these longer intervals. If change is nonlinear in form,^1^ from a simple quadratic curve all the way up to highly complex nonlinear functions (often modeled with generalized additive models), then preferring long intervals between measurement occasions runs the risk of missing those nonlinear patterns of change. While with short measurement intervals, a linear functional form model (e.g., two-time point LCS) can provide a local linear approximation of change, at longer intervals, these models are likely to show more exaggerated misfit to the underlying developmental process (even though the misfit may still go unnoticed in practice and lead to erroneous model selection, see Ghisletta et al., 2020). Fig. 4 displays the issue, where a quadratic curve can be well approximated by nine linear pieces (red), but increasingly long intervals between measurement occasions degrades the ability to approximate the underlying parabolic shape of change.

## 3 Conclusion

Using effective error and effective curve reliability, we show how asymptotic results on the precision of linear growth curve models can be used to assess their ability to recover individual differences in linear slopes. This approach allows for comparing alternative longitudinal study designs under the assumption of linear change, including different number of time points, study duration, indicator reliability and missing data mechanisms. Simulation studies still provide added value when the asymptotic conditions are violated (e.g., small samples, non-negligible intercept-slope correlation), distributional assumptions are violated (non-normal responses), or missingness is non-random. Future work is needed to derive asymptotic estimates for more general cases, such as other shapes of change or medium-to-large intercept-slope correlations. Here, however, we specifically used the asymptotic results to show that the criticisms of the two-time point model by Parsons and McCormick (2024) can be addressed by thoughtful alterations to how we design longitudinal investigations. Linear change can be measured very well with only two measurement points if the measurement instruments are reliable and enough time has passed for individual differences in linear slopes to stand out from measurement noise (Rogosa and Willett, 1983). Note that residual error variance in a univariate LCM is the sum of two components, *slope regression residual variance* and *indicator error variance* (Brandmaier et al., 2018). Slope regression residuals are due to possible misspecification errors in the shape of change, and indicator errors capture measurement error in the observed variable at each occasion. To this end, it is highly recommended to use latent change score and latent curve models with multiple indicators to identify both variance sources and address various problems related to measurement errors in models of change (von Oertzen et al., 2010). Ultimately, linear models may be the wrong choice of model for changes over longer periods of time (Ghisletta et al., 2020), given that the mechanisms that drive maturational, learning-related, or senescent changes typically result in non-linear trajectories at the individual level. If we are interested in veridically capturing such changes, we need four time points or more (Ghisletta et al., 2020, Parsons and McCormick, 2024). And in either the linear or non-linear case, both the number of measurement occasions and the time elapsing between measurement occasions will affect our ability to capture individual differences in linear slopes. Then, a minimum of four time points or more is required to model quadratic or exponential trajectories. Still, one should pay attention to the differential effects of time passing and measuring more often. In sum, we concur with Parsons and McCormick (2024) that precise measurement is key to longitudinal brain imaging studies and that existing studies may often have limited precision. However, we would like to emphasize that the low power in two time-point models observed in their simulations is not an inherent limitation of the model itself but a sub-optimal constellation of study properties as they occur in practice. Indeed, more time points are better. But just waiting longer for individual differences to develop further, may be even better.

## CRediT authorship contribution statement

**Andreas M. Brandmaier:** Writing – review & editing, Writing – original draft, Conceptualization. **Ulman Lindenberger:** Writing – review & editing. **Ethan M. McCormick:** Writing – review & editing, Conceptualization.

## Declaration of competing interest

The authors declare that they have no known competing financial interests or personal relationships that could have appeared to influence the work reported in this paper.

## Footnotes

1 Nonlinear forms can be fit using both truly nonlinear models (where parameters can enter the equations in other ways than simple addition) or linear parameter models which approximate nonlinear change, either through transformations (e.g., 1/age, McCormick et al., 2023) or linearization (Blozis, 2004; e.g., Browne, 1993, McCormick, 2024).
